# The secretion profile of mesenchymal stem cells and potential applications in treating human diseases

**DOI:** 10.1038/s41392-022-00932-0

**Published:** 2022-03-21

**Authors:** Yuyi Han, Jianxin Yang, Jiankai Fang, Yipeng Zhou, Eleonora Candi, Jihong Wang, Dong Hua, Changshun Shao, Yufang Shi

**Affiliations:** 1 Department of Ophthalmology, The Affiliated Hospital of Jiangnan University, 214000 Wuxi, China; 2 The Third Affiliated Hospital of Soochow University, Institutes for Translational Medicine, State Key Laboratory of Radiation Medicine and Protection, Key Laboratory of Stem Cells and Medical Biomaterials of Jiangsu Province, Medical College of Soochow University, 215000 Suzhou, China; 3 Department of Experimental Medicine, TOR, University of Rome Tor Vergata, 00133 Roma, Italy; 4 IDI-IRCCS, 00166 Rome, Italy; 5 Department of Medical Oncology, The Affiliated Hospital of Jiangnan University, 214000 Wuxi, China

**Keywords:** Mesenchymal stem cells, Inflammation

## Abstract

Mesenchymal stromal/stem cells (MSCs) possess multi-lineage differentiation and self-renewal potentials. MSCs-based therapies have been widely utilized for the treatment of diverse inflammatory diseases, due to the potent immunoregulatory functions of MSCs. An increasing body of evidence indicates that MSCs exert their therapeutic effects largely through their paracrine actions. Growth factors, cytokines, chemokines, extracellular matrix components, and metabolic products were all found to be functional molecules of MSCs in various therapeutic paradigms. These secretory factors contribute to immune modulation, tissue remodeling, and cellular homeostasis during regeneration. In this review, we summarize and discuss recent advances in our understanding of the secretory behavior of MSCs and the intracellular communication that accounts for their potential in treating human diseases.

## The identification of MSCs

In 1970, Alexander J. Friedenstein and colleagues described an adherent and non-hematopoietic cell type present in the mouse bone marrow (BM) that could form fibroblast-like colonies in vitro, unlocking the door to the world of mesenchymal stem cells (MSCs).^1^ While MSCs, which are later found to reside in various organs, can generally self-renew and exhibit stromal cell-like characteristics in vitro, the lineages that contribute to MSCs in each organ in vivo and their spatiotemporal changes during development have yet to be well explored. An early study of the hierarchy of BM-derived mesenchymal progenitors showed that Sca1^+^ progenitors can differentiate into CD146^+^ and CD166^+^ progenitors sequentially.^2^ While all three types of progenitors support bone formation, only Sca1^+^ progenitors can home back to the BM through a chemotactic axis post-intravenous infusion. Another report showed that the niches formed by interleukin (IL)-7^+^ mesenchymal progenitors could functionally regulate hematopoietic stem cell maintenance and multilineage differentiation.^3^ These MSCs in BM highly express the intermediate filament protein nestin and are located around hematopoietic stem cells (HSCs).^4^ The nestin^+^ MSCs are proven to regulate the homing of transplanted HSCs to BM,^4^ as well as guiding immune cells to egress to circulation.^5^ In other organs, most of the mesenchymal progenitors are closely associated with capillaries and blood vessels.^6–8^ These perivascular cells display phenotypes similar to those of MSCs derived from BM and dental pulps.^9^ A population of stromal cells that resides among choroidal vascular endothelial cells was also recognized to display the MSC phenotype and possess the capacity for mesenchymal differentiation.^10^ Thus, blood vessel walls in diverse human tissues (such as BM, umbilical cord (UC), adipose, muscle, and placenta) are considered as the primary dwellings of progenitor cells that give rise to MSCs.

The first batch of MSCs during embryonic development could be traced to Sox1^+^ neuroepithelium partly through a neural crest intermediate stage,^11^ arguing for their ectodermal origin. The MSC lineages during organ development are being actively investigated and, owing to the widespread use of single-cell sequencing, imaging analysis, and tracing technologies, functionally distinct new subsets of MSCs are emerging rapidly.

## MSC isolation and characterization

Well-characterized MSCs can now be isolated and propagated in vitro from multiple organs (such as BM, dental pulp, thymus, muscle, pancreas, and lung).^12^ According to the International Society for Cellular Therapy (ISCT)-published minimal guidelines to define human MSC identity, the isolated cells are generally positive for CD105, CD73, and CD90, and negative for CD45, CD34, CD14, or CD11b, CD79α, or CD19 and MHC class II.^13^ Additionally, these cells possess the potential of specific-lineage differentiation toward osteoblasts, adipocytes, or chondrocytes, as well as the capacity of plastic adherence when cultured in vitro.

### Tissue specificity

MSCs isolated from different sources can vary in their gene expression patterns and differentiation potentials.^14^ There are several non-classical markers (such as CD36, CD163, CD271, CD200, CD273, CD274, CD146, CD248, and CD140b) that potentially distinguish MSCs of different sources.^15^ For instance, CD271 is a surface marker for the majority of BM-derived MSCs (MSC(BM)s),^16,17^ while this marker is inadequate for the isolation of MSCs from other sources(such as UC, dental pulp, or placenta).^18^ Lately, a population of highly proliferative multipotent progenitors marked by dipeptidyl peptidase-4 (DPP4)/CD26 has been discovered during the development of subcutaneous adipose tissue in mice through single-cell RNA sequencing analysis. These progenitor cells could produce two subpopulations, committed preadipocytes marked by intercellular adhesion molecule-1 (ICAM-1) and CD142.^19^ More recently, the MSCs/fibroblast atlases were constructed by integrating available single-cell transcriptomic data.^20^ Based on the transcription profiles across tissues, two universal subtypes were identified as the primitive lineages to generate more specialized descendants in health and disease.

Since freshly isolated MSCs comprise multiple MSC subgroups or progenitors varying in proportions depending on the source of origin, MSCs with different tissue origins may meet specific needs. For example, MSC(AD)s are more efficient in supporting hematopoiesis and angiogenesis than MSC(BM)s.^21^ Human UC-derived MSCs (MSC(UC)s) exhibit a relatively more stable capacity of proliferation and trilineage differentiation than MSC(BM)s.^22^ Compared to MSCs from BM, adipose tissue, or placenta, MSC(UC)s possess the strongest potential to suppress T lymphocyte proliferation by inducing cell-cycle arrest (G0/G1 phase) and apoptosis, along with altered expression of apoptosis-related genes.^23^

### Cell plasticity

The phenotype and biological features of MSCs could be dynamically altered by culture conditions, leading to distinct capacities of differentiation and proliferation during their expansion in vitro. Freshly isolated MSC(BM)s from humans and mice lack the expression of CD44 but display poly directional differentiation potential.^24^ During in vitro expansion, freshly isolated MSC(BM)s acquire CD44 expression without compromising their proliferation efficiency or differential potential, accompanied by dramatic upregulation of hyaluronan synthases (HAS1 and HAS2), growth factors, and matrix proteins.^24^ On the other hand, young MSCs undergo cellular senescence in response to transforming growth factor-β (TGFβ2), while anti-TGFβ antibodies could reverse the aging phenotypes of old MSCs.^25^ In addition, peritoneal dialysis effluent-derived MSCs (MSC(P)s) displayed a homogeneous pattern of classical MSC markers with multipotency in vitro, which was decided by specific culture medium.^26^ MSC subsets with distinct phenotypic and functional properties could be furtherly identified by using markers including CD56 and MSC antigen-1 (MSCA-1).^27^ Nestin marks human MSC(BM)s that are more readily differentiated to insulin-producing cells (IPCs) than the nestin^−^ cells.^28^ They highly express maintenance genes and favor BM homing of HSCs.^4^ CD271^+^ adult MSCs show higher clonogenic and osteogenic capacities than CD271^−^ ones.^29^ The Thy-1(CD90)^−/−^ MSCs are unable to form healthy bone tissues as the wild-type counterparts do and are more likely to differentiate into adipocytes.^30^ On the contrary, the inflammatory cytokine lipocalin-2 is shown to augment the transcription of osteogenic genes in MSCs, exacerbating the cascade of dysregulated cellular events in myelofibrosis.^31^ Revealing this high plasticity of MSCs has opened new perspectives to explain the disrupted balance of adipogenesis and osteogenesis in developmental and metabolic diseases.

### Therapeutic applications of MSCs

**Fig. 1:**
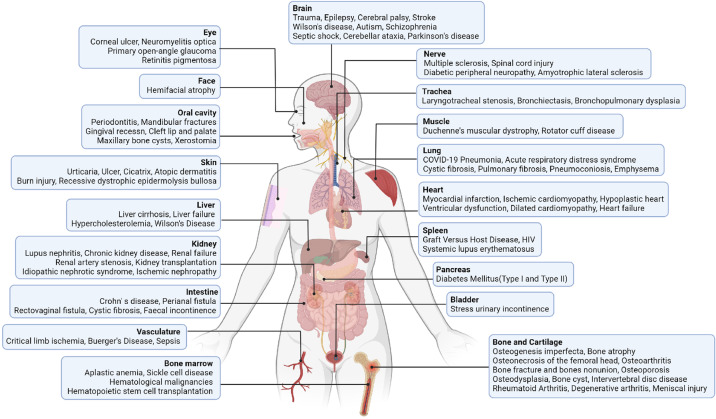
MSC-based clinical trials involve a variety of diseases in different organs and tissues. MSC-based clinical trials are mainly applied to the diseases associated with inflammation, wound healing, infection, as well as degeneration in diverse organs and tissues. The figure shows the types of diseases that have completed clinical trials (reproductive diseases and aging are not listed), and the most widely applied diseases involve the bone and nervous system. MSCs possess a strong capacity in balancing immune responses, especially in autoimmune disorders, such as GvHD and Crohn’s disease. As a lot of refractory diseases are often combined with poor repairing of damaged tissues and dysfunction of diseased organs, such as bones nonunion and multiple sclerosis, the clinicians also favor the multi-directional differentiation potential and pleiotropic effects of MSCs, to promote wound healing and functional recovery. In addition, researchers have been gradually investigating the therapeutic potential of MSC-based therapy in some congenital diseases. (Created with BioRender.com)

Researchers were firstly attracted by the self-renewal capacity of MSCs and their differentiation potential towards multiple lineages, afterwards the ability of MSCs to regulate immune responses was discovered. These biological properties of MSCs promoted the development of therapeutics for tissue regeneration. MSC(BM)s, as one of the main supporters for hematopoiesis, could restore defective BM microenvironment for myelopoiesis. They are also involved in lymphocyte maturation^32,33^ and integrate with the inordinate immune system to modulate tumor progression, such as reprogramming host macrophages to retard leukemia development.^34^ Both allogenic or autologous MSCs are able to traverse the circulation through the chemotactic network and migrate to specific destinies to support the growth or function of resident cells in the lesion sites. This mobility feature and the low immunogenicity endow MSCs with biological acceptability in vivo. Strikingly, MSC(BM)s can greatly inhibit the immune responses mediated by active lymphocytes in a dose-dependent manner.^35^ This regulatory potential of MSCs has attracted much attention and has been shown to be surprisingly effective in controlling inflammation and balancing immune response. Allogenic MSCs were shown to promote orthopaedical repair,^36^ skin wound healing,^37^ and nerve regeneration/reconnection.^38^ The clinical superiority of MSCs in treating inflammatory and degenerative diseases has been intensively reported. As of November 25, 2021, a total of 965 mesenchymal stromal/stem cell-based, clinical trials had been registered in the US National Institutes of Health (https://clinicaltrials.gov/), including ongoing, withdrawal, complete and unknown status studies (Table 1). Apparently, the MSC-based clinical trials are mainly applied to inflammation, wound healing, infection, organ dysfunction, as well as degenerative diseases in different organs and tissues (Fig. 1). However, several pitfalls of engrafted MSCs are encountered during practical use, such as their limited vitality,^39^ uncertain responsiveness, as well as the difficulty in monitoring their differentiation in situ. The massive expansion of MSCs in vitro also incur high cost, which cannot be afforded by many financially strapped patients, especially when taking the transient curative effect into consideration. A shift in focus from MSCs allografting to the effector molecules that mediate the cell-specific effects should bypass the disadvantages resulting from immune compatibility, tumorigenicity, and the unpredictable pathogen carried by living cells, in addition to the pitfalls listed above.^40^

**Table 1:** Status of MSC-based clinical trials for various diseases registered at NIH.gov

	Open studies				Closed studies				Unknown status
Diseases	Recruiting	Enrolling by invitation	Not yet recruiting	Active, not recruiting	Completed	Suspended	Terminated	Withdrawn	
Hematological	4	0	0	0	8	0	2	2	17
Cardiovascular	9	1	7	5	19	2	5	3	10
Renal	6	0	3	3	6	0	2	1	11
Hepatic	7	0	3	1	10	1	1	0	34
Respiratory	39	1	19	13	19	1	3	2	15
Cutaneous	6	2	5	3	9	1	1	0	10
Neural	31	5	7	15	66	6	7	8	34
Skeletal	30	1	10	3	58	3	4	8	34
Muscular	4	0	1	2	4	0	1	1	5
Diabetes	14	1	1	4	9	0	1	1	25
GvHD	6	0	2	1	11	0	1	2	16
Crohn’s	8	0	1	0	8	0	0	1	6
SLE	2	0	3	0	3	0	0	0	6
Other	29	4	9	10	53	1	3	3	40
Total	195	15	71	60	283	15	31	32	263

*GvHD* graft versus host disease, *SLE* systemic lupus erythematosus.

## The secretory functions of MSCs

MSCs engraftment is usually limited by the rigorous tissue structure around the injured site, where allografted cells undergo cell death or be engulfed by resident cells. How MSCs exert their plerosis and immunoregulatory effects have been intensively studied. In the skin incison, the supernatant of cultured MSCs not only enhanced the function of keratinocytes and endothelial cells, but also attracted macrophages into wound healing process.^41^ Considering that the paracrine function of MSCs plays a major role in tissue repair, there is a renewed interest in the components and molecular basis of MSCs secretion involved in the interaction between allogeneic cells and the tissue microenvironment.

### Secretion is central to MSC-based therapy

Our early studies demonstrated an indispensable role of soluble factors in the immunosuppressive functions of MSCs.^42^ Of note, intrathecal injection of MSCs showed high efficacy than intravenous administration in treating multiple sclerosis, indicating that cells delivered in closer proximity to the damaged areas could bring a higher proportion of trophic factors and immunomodulatory molecules to the lesion sites in central nervous system.^43^ Numerous studies have indicated the paracrine actions of MSCs in treating diseases (Table 2). MSCs-generated signaling molecules can be isolated and enriched as cell-free products in clinical translation. Apart from the essential role of bioactive fractions released by MSCs in modulating the intrinsic tissue repair process, MSCs also express a wide array of chemokines and receptors to form a subtle chemotactic network in vivo for guiding circulating cells to the injury sites, or mobilizing immune cells in inflammatory tissues.^44^ Importantly, the tissue remodeling process mediated by MSCs cannot be attributed to a single effector, but the combined regulation of various factors to maintain homeostasis. A deeper understanding of the secretion function of MSCs in both physiological and pathological conditions is necessary for designing more effective and safe treatment strategies.

**Table 2:** Outcomes of MSC treatments through paracrine mechanisms

Organ injury and diseases		
Tissue	Target cells/tissues	Outcome
Hair	Dermal papilla cells	Promote hair growth and elongation of hair shafts^251^
Skin	Cutaneous tissue	Ameliorate *Psoriasis vulgaris*^252^
	Dermal fibroblast, keratinocyte	Accelerate skin wound closure and reduce inflammation^253,254^
Nose	Immune cells	Reduce allergic rhinitis^255^
Eye	Limbal myofibroblasts, neutrophils	Anti‐inflammation and reduce cornea fibrosis^256^
	Retinal endothelial cell, microglia	Anti-inflammation and modulate neurovascular in retina^257^
	Immune cells, vascular endothelial cells	Alleviate allergic conjunctivitis^258^
Heart	Cardiac tissue	Improved arrhythmias and reduced cardiac fibrosis^259^
	Cardiomyocytes	Reduce myocardial ischemic damage^260^
	Cardiac fibroblasts	Promote cell survival and reduce collagen deposition^261^
Intestines	Immune cells	Reduce inflammation in colitis^262^
Liver	Hepatic stellate cell	Reduce liver fibrosis^131^
	Hepatocytes	Promote cell survival and hepatic regeneration^263,264^
Lung	Pulmonary tissue	Alleviate bronchopulmonary dysplasia^265^
	Bacteria	Reduce pneumonia^266^
	Lung fibroblasts	Promote cell survival and restore cell function^267^
	Epithelial cells, fibroblasts	Reduce pulmonary fibrosis^132^
	Epithelial cells	Stimulate functional and structural maturation of the fetal lung^268^
Kidney	Renal tubular epithelial cells, immune cells	Reduce inflammation and attenuate renal fibrosis^269–271^
	Immune cells	Reduce inflammation and promote renal injury repair^272^
Nerve	Nerve fibers	Reduce neuroinflammation and ameliorate degenerative changes^273^
	Neurons and Schwann cell	Reduce neuroinflammation and promote cell survival^274^
	Neural stem cells, neurite	Promote neuronal differentiation and neurite outgrowth^275^
	Neural cells and myelin	Modulate immune response and myelin repair^133^
Bone	Skeletal tissue	Facilitate bone repair^276,277^
	Joint, cartilage, synovium	Reduce inflammation^278,279^
	Chondrocyte, cartilage matrix	Preserve bone microarchitecture and promote cell survival^280^
Muscle	Muscular tissue	Promote skeletal muscle regeneration^281^
	Muscle cells	Prevent muscle atrophy^282^
Reproduction	Testicular tissue	Promote cell survival and protect spermatogenesis^283^
	Ovarian tissue	Reduce ovarian injury and improve ovarian function^284^
*Systemic disorders*		
Diabetes	Pancreatic islets, immune cells	Reduce inflammation and preserve pancreatic function in type I diabetes^285^
	Hepatocytes, immune cells	Promote cell survival and reverse insulin resistance in type 2 diabetes^286^
Obesity	Adipose tissue	Increase adiponectin secretion and multimerization^287^
Atherosclerosis	Vascular, immune cells	Reduce macrophage accumulation and regulate M2 polarization^288^
GvHD	Immune cells	Suppress immune response^289^
Sjögren’s syndrome	Immune cells	Enhanced the suppressive function of myeloid-derived suppressor cells^290^

### Variability of the secretion profiles

#### Orchestration by inflammatory signals

There are various types of inflammatory factors in the process of tissue damage, repair, and remodeling along with disease progression, these factors are indispensable for boosting the reaction of MSCs. One of the innate responses to acute injury is the formation of a relatively hypoxic microenvironment in situ, which is resulted from the increase in oxygen demand of infiltrated cells and the high metabolic rate, coupled with the vasoconstriction caused by inflammatory stimuli.^45^ Hypoxia rapidly upregulates the level of ICAM-1 in inflamed sites (such as the endothelium) via hypoxia-inducible factor 1α(HIF1α)^46^ and ICAM-1 could remarkably promote MSC migration to inflamed tissues.^47^ Also, the paracrine property of MSCs to release chemotactic and angiogenic factors is significantly amplified under hypoxic condition.^48^

**Fig. 2:**
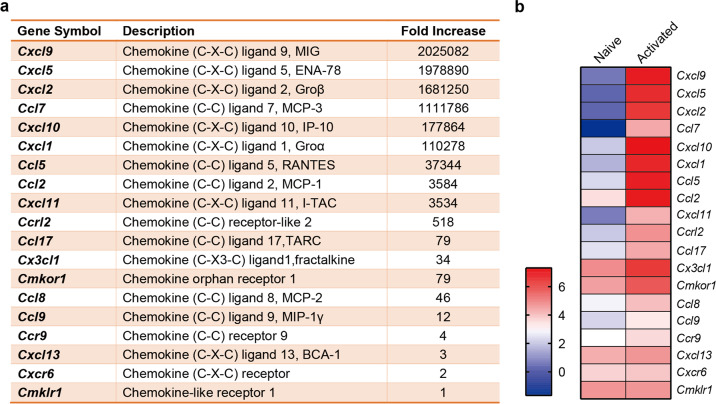
Supernatant of anti-CD3-activated splenocytes increased the gene expression of chemotactic factors in MSCs. **a** Fold increase of gene expression in the MSCs treated with supernatant from anti-CD3-activated splenocytes, relative to the ones treated by supernatant from naive splenocytes. **b** Heatmap for log value of chemotactic genes expression in MSCs. (Modified from Ren et al., Cell Stem Cell, 2008)

Given that the MSCs stimulated by interferon-γ (IFNγ) and tumor necrosis factor-α (TNFα) or IL-1 exhibited greater immunosuppressive capacity by upregulating the expression of ICAM-1 and vascular cell adhesion molecule-1(VCAM-1) both in vitro and in vivo,^49^ the inflammatory environment is pivotal in shaping the regulatory role of MSCs. During inflammation, the M1 macrophages or T helper cell type 1 (Th1) lymphocytes secrete high levels of proinflammatory cytokines, which confer MSCs dramatic immunomodulatory ability.^50^ We found that inflammation primed-MSCs secreted a series of chemokines to attract immune cells (Fig. 2), and produced inducible nitric oxide synthase (iNOS) in rodents or indoelmine-2–3-dixoygenase (IDO) in other mammalian species to suppress T cell responsiveness.^51,52^ Regarding proliferation, the activated lymphocytes and MSCs are mutually inhibitory in co-culture.^42^ Although the differentiation capacities and immune regulatory functions of individual MSC clones are heterogeneous, priming MSCs with pro-inflammatory agents uniformly amplified their inhibitory effects on T cell response and eliminated the difference in the suppressive extent among different MSC clones.^53^ The inflammatory signals polarize MSCs towards an anti-inflammatory and pro-trophic phenotype for tissue recovery.^54^ In turn, the factors released from MSCs promote the remodeling of damaged tissue microenvironment.^52^

Hyper-inflammation endows MSCs with unique regulatory functions, while low doses of inflammatory cytokines could hardly elicit such a response or even cause an opposite effect. High levels of cytokines stimulate MSCs to inhibit immune responses, whereas MSCs activated by the weak stimulus are barely immunosuppressive but still can release chemokines for attracting immune cells.^55^ The MHC class II expression in MSCs requires stimulation by a low dose of IFNγ, which endows MSCs with antigen-presentation effect to enhance T cell-mediated immune response.^56,57^ When the IFNγ levels increase to a level higher than 25 pg/ml, MSCs become immune-suppressive with diminished antigen-presentation cell (APC) function.^56^ Signal transducer and activator of transcription (STAT) 1 and STAT3 are both activated by IFNγ at early stages. Inhibition of mTOR further promotes pSTAT1 nuclear translocation and strengthens the ability of MSCs to inhibit T cell vitality. However, sustained exposure to IFNγ led to inhibition of STAT3 activity and impaired the capacities of proliferation and differentiation of MSCs.^58^

#### Tumor-associated MSCs

MSCs can sense distinct signals from surrounding tissues to adapt to different pathological conditions.^59^ In comparison with naïve MSCs, tumor resident MSCs produce more chemokines that recruit monocytes/macrophages to the tumor tissue.^60^ The tumor microenvironment triggers the transformation of MSCs into cancer-associated-MSCs (MSC(CA)s), which in turn polarize monocytes to a pro-tumoral phenotype.^61^ Tumor cells, such as those of multiple myeloma, can deliver miR-146a-containing exosomes into MSCs. This microRNA has been shown to elevate the secretion of several cytokines and chemokines in MSCs to support tumor metastasis.^62^ The acute myeloid leukemia (AML) cells highly expressed macrophage migration inhibitory factor (MIF) to activate its receptor CD74 on MSC(BM)s and thereby promoted MSC(BM)s to secrete IL-8 to support tumor cells survival.^63^ MSC(BM)s can also express periostin to increase the C-C motif chemokine ligand (CCL)2 expression in acute lymphoblastic leukemia (ALL) cells, which conversely upregulates periostin expression in MSC(BM)s and contribute to leukemia progression.^64^ Thus, there is a crosstalk between MSCs and the surrounding factors in the tumor microenvironment.

Despite that MSCs are reported to promote survival of cancer cells,^65–67^ discerning the specific role of MSCs in tumorigenesis is critically needed for targeted cancer therapy. MSC(CA)s support the growth and invasion potential of cancer cells through secreting cytokine GM-CSF, providing a novel cytokine pathway for therapeutic intervention.^68^ During traditional ROS-inducing chemotherapies for ALL, MSC(BM)s from patients could be activated by cytarabine and rescue the stressed cancer cells through mitochondrial transfer. Thus, steroids and microtubule inhibitors can be exploited to improve the therapeutic strategies for ALL, by preventing MSC activation and disrupting microtube formation respectively.^69^ In another study, promyelocytic leukemia gene expression in MSCs was found to play an essential role in generating pro-inflammatory cytokines and soluble factors, which are crucial to the persistence of leukemic cells in patients with chemotherapy resistance.^70^ C-X-C motif chemokine ligand (CXCL)12-expressing MSC(BM)s are also important for retaining quiescence leukemic stem cells (LSCs), while MSC-specific deletion of CXCL12 makes LSCs become more sensitive to tyrosine kinase inhibitors, although at the risk of leukemic cell expansion.^71^ On the other hand, survival of leukemia-bearing mice could be prolonged by intra-BM transfusion of MSC(BM)s from healthy mice. The healthy MSC(BM)s functionally recovered the host MSCs and reprogrammed the host macrophages in BM to execute the tissue repair function.^34^ These investigations strongly suggest that targeting MSCs in tumors may overcome major obstacles of drug resistance and disease recurrence.

### Context-dependent efficacy of MSC therapies

The recognition of the paracrine mode of MSCs opens a new venue for understanding the cellular mechanisms of MSC therapies for diverse diseases. It was reported that toll-like receptor 4 (TLR-4) activated MSCs increased VCAM-1 and ICAM-1 dependent binding of leukocytes, whereas TLR3 stimulated MSCs exhibit enhanced affinity to leukocytes through hyaluronic acid (HA).^72^ Therefore, specific stimuli are important in provoking the immunomodulatory capacities of MSCs during MSCs-based therapy. As the inflammatory signals always fluctuate with the progression of diseases, various immunosuppressive signals in the tissue microenvironment, including TGFβ and IL-10, as well as the immunosuppressant such as cyclosporin A, have been proven to abrogate MSCs-mediated immunosuppression and to sustain inflammation instead.^59^ Injection of MSC(AD)s at the peak of experimental autoimmune encephalomyelitis (EAE) produced more satisfactory effects than those injected during the regression phase.^73^ Moreover, a clinical trial of autologous MSC transplantation yielded improved clinical scores in multiple sclerosis patients with active disease progression.^43^ The use of steroids may complicate the therapeutic effect of MSCs. Steroids disrupt STAT1-mediated expression of IDO and iNOS and therefore may cause a dose-dependent reversion of MSCs mediated-T cell suppression without affecting the chemokines induction.^74^

Additionally, the secretion spectrum of MSCs is not only influenced by diverse exogenous stimuli, but also by the status of MSCs themselves. MSC(AD)s have a higher immunomodulatory capacity than MSC(BM)s at equal cell numbers, at least partly because of higher expressions of TGFβ than MSC(BM)s with their vigorous metabolic activity.^75^ The mitochondria of MSC(AD)s from atherosclerotic patients possess a higher level of reactive oxygen species and altered secretion profile, leading to an impaired immunosuppressive capacity.^76^ In the organoid model of alveolospheres, aged lung MSCs have higher levels of NADPH oxidase 4(NOX4) to produce oxidants and acquire senescence-associated secretory phenotype, so that they lose the normal 3D structure with type 2 alveolar epithelial cells.^77^ The senescent MSCs appeared to be less potent in tissue protection than the young ones, due to insufficient production of growth factors and chemokines.^78^ Therefore, senescent MSCs deploy a more blunted secreting response to the activated immune cells compared to young MSCs, but IFNγ could partly restore the immunosuppressive deficiency of senescent ones.^79^

Collectively, these discoveries suggest that MSCs are highly plastic in their secretion spectrum. The context of pathological status in vivo and MSCs-produced mediators vary dependently and thus result in heterogeneous immunoregulatory functions, which could partly explain why clinical trials of MSC applications have produced ambiguous outcomes.^80^ Thus, it is essential to tailor MSCs secretion to meet the context-specific needs.

## Major factors released by MSCs

The secretory profiles of MSCs encompass a variety of biologically active ingredients. A large portion of bioactive factors is packaged by vesicles for external transmission. Most molecules are discharged outside the cell through the classical exocytosis fusion mechanism, while the other transportation involves direct membrane translocation of proteins.^40^ In fact, there are few reports about the mechanisms of intracellular molecule assembly and transmembrane transport process in MSCs.

### Extracellular vehicles

#### Generation and actions

One of the most important mechanisms for MSCs to communicate with other cells is through extracellular vesicles (EVs). Exosomes are the smallest subtype of EVs that have been intensively studied. Exosomes generally originate from endosomes, as their membranes are enriched in lipids rafts, which are involved in the fusion and release cascades between intraluminal vesicles (ILV) and multivesicular bodies (MVB).^81^ The fusion of MVB with the plasma membrane leads to the release of exosomes. Exosomes may subsequently be taken up by other cells via membrane fusion, endocytosis or cell-type-specific phagocytosis.^82^ Microvesicles (MVs) are slightly larger than exosomes and are formed from plasma membrane budding and fission. TNFα upregulates Fas and Fas-associated phosphate-1(Fap-1) expression via the NF-κB pathway and facilitates the Fas/Fap-1/Caveolin-1 complex transfer to the cell membrane of MSCs derived from the gingiva, promoting membrane fusion to release small EVs in a soluble N-ethylmaleimide-sensitive factor (NSF) attachment protein receptor (SNARE)-dependent manner.^83^

The nanoscale exosomes can easily shuttle through tissues and biological barriers to transfer microRNAs, lipids, and proteins, thus they have been adapted as therapeutic agents.^84^ For example, exosomal miR-125b-5p was shown to mediate the therapeutic effects of MSCs in myocardial infarction and ischemic acute kidney injury, it directly downregulated p53 and prevent cell apoptosis through reducing autophagic flux or cell cycle arrest.^85–87^ In practice, the immunoregulatory functions of MSCs partially rely on exosomes, which mediate the MSC-immune cell crosstalk in some pathological conditions. The exosomes from MSC(AD)s inhibit T cell proliferation, differentiation, and activation as well.^88^ MSC-derived exosomes were shown to ameliorate the pathological changes of experimental autoimmune uveoretinitis by preventing the accumulation of inflammatory cells (CD4^+^T cells, neutrophils, NK cells, and macrophages) around the eyes and reducing the percentage of CD4^+^IFN-γ^+^ and CD4^+^IL-17^+^ cells in the retina, without inhibiting proliferation of IRBP-specific T cells.^89^ The mitochondria of donor MSCs, carried by EVs, can be transferred to neighboring macrophages to enhance oxidative phosphorylation, consequently achieving an anti-inflammatory and highly phagocytic macrophage phenotype.^90^ MSC(AD)s transferred the exosomes loaded with active STAT3 into macrophages and polarized them towards the anti-inflammatory M2 phenotype through the transactivation of arginase-1. These M2 macrophages reversely promoted MSC(AD) proliferation and lactate production, thus facilitating metabolic activity and resistance to obesity progression.^91^ Exosomal miR-182 delivery from MSCs to macrophages directly downregulates TLR-4 to confer M2 phenotype, contributing to the therapeutic effects of exosomes on myocardial ischemia/reperfusion injury.^92^ Moreover, MSC-derived MVs also enhanced monocyte phagocytosis of bacteria in severe pneumonia and ameliorated inflammation in injured alveolar epithelium.^93^ Notably, EVs can attach to the extracellular matrix (ECM), and the soluble factors released from EVs can also attach to ECM or directly act on adjacent target cells. Intravitreal injection of MSC-derived EVs significantly enhanced functional recovery, and decreased neuroinflammation and apoptosis in retinal ischemia.^94^ The MSC-EVs could bind to vitreous humor components and persist in the vitreous humor for a long time to provide sustainable protection.^94^ The therapeutic efficacy of MSC-derived exosomes in retinal degeneration models was long-lasting, as the protective effects on photoreceptors and retina could be detected even months after a single injection.^95^

#### EV-based therapeutics

EVs have emerged as a rising therapeutic paradigm for cell-free MSC-based therapies. Clinical study has shown that the use of MSC-EVs led to significant improvement in GvHD symptoms, remarkably reducing the dosage of steroids.^96^ MSC-EVs induced polarization of M2 macrophages to dampen inflammatory response in damaged tissue sites, thereby promoting tissue remodeling in diabetic wounds.^97^ The MSC-EVs were also reported to ameliorate lung injury^90^ and decelerate renal fibrosis via modulating the phenotype and function of infiltrated macrophages.^98^ MSC-EVs act as efficiently as MSCs in treating various degenerative diseases and immune dysfunctions, while EVs bypass a series of drawbacks of direct cell infusion. The therapeutic efficacy of human MSCs can be reproduced by the administration of their autologous exosomes.^99^ Moreover, genetic engineering modified MSC-derived exosomes can act as ‘Trojan horses’ to target the tumor microenvironment and strengthen tumor immunotherapy.^100^ Such exosomes are constructed to carry galectin-9 siRNA and oxaliplatin pro-drug. More importantly, in sharp contrast to native MSCs, their EVs do not cause undesired immune responses due to fewer stimulatory HLA-complex molecules and surface co-stimulators.^101,102^ In COVID-19 treatment, MSCs administration may potentially cause coagulopathy, which synergizes with COVID-19 pneumonia and deteriorates the patients’ condition. In comparison, low immunogenic exosome delivery can avoid this side effect.^103^

However, how to make EV-based therapies more practical and effective in disease treatment is an urgent issue in the clinical application of MSCs. EVs from different origins of MSCs may have conflicting effects, as the exosomes released by MSCs of Langerhans islets in non-obese diabetic (NOD) mice have been shown to be highly immunostimulatory and able to trigger autoimmune response.^104^ Different isolation methods determine the yield and purity of exosomes.^105^ Distinct culture conditions, such as oxygen concentration and culture matrix, also influence the functional properties of EVs. Exosomes derived from MSCs preconditioned with hypoxia conditions possess greater therapeutic effects on bone fracture healing. In this scenario, the activation of HIF-1α increased exosomal miR-126 abundance, consequently promoting the proliferation, angiogenesis, and migration of endothelial cells through the SPRED1/Ras/Erk signaling pathway.^106^ Certain biomaterials combined with EVs can reinforce stem cell-based tissue repair. MSC-derived exosomes loaded in a 3D printed cartilage ECM/gelatin methacrylate bio-scaffold have improved osteoarthritis therapeutic efficacy.^107^ The EV-based therapy may provide a new venue for the treatment of diseases, but the variability in the contents of EVs due to harvest procedures and cell sources may complicate their clinical applications.

### Chemokines and receptors

#### MSCs homing

Like leukocytes, MSCs can fully transverse the vascular endothelium, a process that can be significantly enhanced by the presence of CXCL9, CXCL16, CCL20, and CCL25.^108^ MSCs harness the network of multiple chemotactic factors to access specific locations and further facilitate tissue remodeling in situ. In this review, we distill the recent studies about the intercellular activities mediated by the wide set of chemotaxis (Table 3). Among these chemotactic gradients, CXCL12(SDF1) is the most prominent one for accumulating stem cells in the BM. The CXCL12/ C-X-C motif chemokine receptor (CXCR)4 axis is essential for MSCs migration, homing, and engraftment in BM stroma,^109^ and is vital for sustaining the function and development of other precursor cells in tissues. For instance, MSCs modulate the self-renewal and the growth of cardiac cKit^+^ cells via the CXCL12/CXCR4 pathway.^110^ Intrinsic expression of CXCR4 is required for the differentiation of lymphoid precursors and their positioning adjacent to the mesenchymal progenitors in the BM, whereas CXCL12 deletion causes a decrease in natural HSCs and expansion of abnormal HSCs.^3^ CXCL12 is one of the target genes of HIF-1α, which is rapidly upregulated by ischemia or reduced oxygen tissue tension in the initial stage of acute injury. The mobilization of MSCs could be propagated by the hypoxic conditions through induction of the CXCL12–CXCR4 axis.^111^ Moreover, the chemotactic function of CXCL12 could be augmented by many priming agents such as complement components^112^ (C1q) and bioactive lipids^113^ (sphingosine-1 phosphate, or ceramide-1 phosphate). TNFα signal also stimulates MSC migration towards the inflammatory site in a dose-dependent manner.^114^ Therefore, the bioactive gradients released from damaged tissue amplify and shape the chemokine network of MSCs.

**Table 3:** The chemotactic axis involved in MSC-mediated efficacy

MSC-secreted ligands	Target cell/tissue	Receptors	Effects
CCL2	Macrophages	CCR2	Macrophage polarization^291^
CCL3/4	Colorectal cancer cells	CCR5	Tumor progression^292^
CCL5	Breast cancer cells, colorectal cancer cells	CCR1/5	Tumor metastasis^293,294^
CCL20	CD4^+^ T cells	CCR6	Lymphocyte recruitment and MSC differentiation^295^
CCL21	Melanoma, glioma, lung carcinoma cells	CCR7	Tumor metastasis^296^
CXCL1	Multiple myeloma cells	CXCR2	Tumor metastasis^62^
CXCL1/2/8	Macrophages	CXCR1/2	Macrophage polarization and tumor progression^61^
CXCL1/5	Mammary cancer cell	CXCR2	Tumor metastasis^297^
CXCL8	Acute myeloid leukemia cells	CXCR1/2	Acute myeloid leukemia cells survival^63^
CXCL8	CD4 ^+^ T cell	CXCR1/2	CD4 + T cell migration^298^
CXCL12	Cardiac myocytes	CXCR4	Progenitors recruitment and myocyte survival^299^
CXCL12	Cardiac cKit^+^ cells	CXCR4	Cardiac cKit+ cells migration and prolferation^110^
CXCL16	Gastric cancer cells	CXCR6	Tumor progression^300^
CX3CL1	Microglia	CX3CR1	Neuroprotective phenotype of microglia^301,302^

MSC-expressed receptors	Donor cell/tissue	Ligands	Effects
CCR1	Macrophages	CCL3	MSC migration^303^
CCR2	Macrophages	CCL2	MSC migration^303^
CCR1	Hepatoma cells	CCL15	MSC migration^304^
CCR4	Bone marrow	CCL22	MSC transendothelial migration^305^
CCR6	Hepatoma cells	CCL20	MSC migration^304^
CCR7	Intradermal site	CCL21	MSC migration and wound repair^306^
CCR9	Myeloma	CCL25	MSC mobilization^307^ and tumor progression^308^
CCR10	Skin	CCL27	MSC recruitment^309^
CXCR2	Buccal mucosa	CXCL2	MSC migration and accelerate ulcer healing^310^
CXCR2	NK cell	CXCL7	MSC recruitment^311^
CXCR5	Injured sites	CXCL13	MSC recruitment^312^
CXCR6	Bone marrow	CXCL16	MSC transendothelial migration^305^

#### Actions on immune cells

The decisive roles of chemokines and receptors expressed by MSCs in cell mobilization have been extensively investigated. Additionally, they are also defined as the driving force to regulate the migration of immune cells, so that MSCs functionally foster the immune response to maintain homeostasis in the body. As illustrated earlier, MSC(BM)s secrete CCL2 in response to TLR ligands or bacterial infection to induce monocyte emigration to the circulation, thereby enhancing resistance to bacterial infections.^115^ The chemotaxis mediated by cues from MSCs assists in recruiting T cells for Fas ligand (FasL)-mediated apoptosis and diminishing excessive inflammatory reactions in treating autoimmune disorders, such as systemic sclerosis and colitis.^116^ The chemotactic gradients have a short half-live and undergo degradation by extracellular proteases. Unlike the neurotoxicity of cleaved-CXCL12 fragments,^117^ the proteolytic processing of CCL2 by matrix metalloproteinase (MMP) generates an antagonistic derivative that inhibits the activity of CD4^+^Th17 cells.^118^ Thus, MSC-derived CCL2 inhibits CD4^+^ T cell activation by suppressing STAT3 phosphorylation and reversing symptomatic neuroinflammation in experimental autoimmune EAE.^118^ Moreover, blockade of CXCR3 or C–C motif chemokine receptor (CCR)5 abolished the MSCs-induced immunological suppression of lymphocytes.^51^ The upregulated CCL5 from irradiated MSCs, as a result of the activation of the cGAS-STING signaling pathway, is responsible for increasing tumor metastasis in mice, by recruiting macrophages to the lung.^119^ Importantly, MSC-mediated immunosuppression in vivo is closely associated with the polarization of tissue-resident macrophages to the anti-inflammatory phenotype. Different from the direct signaling pathway mediated by other immunoregulatory factors, MSC-derived CCL2 requires heterodimerization with CXCL12 to synergistically polarize macrophages via CCR2.^120^ The heterodimerization occurs between members of CXC and CC subfamilies and dramatically alters responsive cell functionality other than mere chemotaxis.^120,121^ The studies above indicate the requirement of chemotactic gradients in MSC-mediated immunoregulatory effects, but the role of chemokines goes well beyond these effects. MSCs release C-X3-C motif chemokine ligand 1 (CX3CL1) to target the C-X3-C motif chemokine receptor 1(CX3CR1) on microglia to control their activation and phagocytosis.^122^ The CX3CR1^+^ microglia are functionally enhanced by an MSC-driven increase in intracellular calcium concentration and display enhanced phagocytotic activity in swallowing axon fragments or apoptotic cell bodies. As expected, MSCs switch microglia to a neuroprotective phenotype and provide a beneficial environment for the regeneration of nerve axons.

### Growth factors

A variety of growth factors have been identified among the secretion profile of MSCs, including vascular endothelial growth factor (VEGF), basic fibroblast growth factor (bFGF), keratinocyte growth factor (KGF), insulin-like growth factors (IGF-1 and IGF-2), and hepatocyte growth factor (HGF). These growth factors are not only important effector molecules that promote tissue repair but can also regulate the differentiation and function of MSCs themselves. VEGF-C induces the phosphorylation of VEGF receptors (VEGFR2, VEGFR3) and the activation of ERK signaling in MSCs. VEGF-C enables MSCs to acquire enhanced expressions of osteogenic marker genes such as RUNX family transcription factor 2 (RUNX2) and facilitates MSC mineralization.^123^ When stimulated by bFGF, the HGF expression in MSCs was upregulated through the JNK signaling pathway, contributing to the tissue repair and suppression of fibrogenesis.^124^

#### Pro-and anti-angiogenesis

The superiority of MSCs in promoting wound healing mainly results from a series of mitogenic and vascular trophic factors, including angiogenic factors to restore the blood supply in ischemia tissues.^125^ It has been confirmed that growth factors (VEGF, HGF, and IGF-1) are rich in MSC culture medium (MSC-CM) and provide a renal protective effect in acute kidney injury after MSCs infusion.^126^ We reported that MSCs treated with TNFα and IFNγ could secret a large amount of VEGF-C that accelerates wound closure through promoting angiogenesis.^127^ Nevertheless, the MSCs-mediated therapeutic effect does not always result from angiogenesis. We found that human MSC(AD)s could effectively inhibit neovascularization and reduce the opacification of ethanol-injured cornea via promoting the clearance of neutrophils during the granulation stage.^128^ Likewise, another study demonstrated that MSCs extracted from corneas and then embedded in fibrin gel for local application prevented corneal neovascularization after corneal injury in mice. These effects were significantly abrogated by knocking down the pigment epithelium-derived factor (PEDF) expression in cornea-derived MSCs (MSC(C)s). Mechanistically, the secretion of anti-angiogenic factors including soluble fms-like tyrosine kinase receptor (sFLT)-1 and PEDF in MSC(C)s attenuated the injury-triggered angiogenesis.^129^

#### Tissue remodeling

MSC-EVs improve the survival of animals with experimental lung injury in part through the secretion of KGF^93^ and HGF,^130^ which is associated with decreased endothelial permeability and protection of cell growth. Mass spectrometry analysis showed that the milk fat globule-EGF factor 8 (MFGE8) secreted by MSCs strongly inhibited hepatic stellate cells and thus prevented liver fibrosis.^131^ Of note, HGF is a crucial factor in MSC-mediated protective effects in chronic inflammatory disease models. HGF does not only prevent epithelial cells from apoptosis but also exhibits anti-fibrotic effects in the experimental fibrosis model.^132^ Moreover, HGF contributes to MSC-mediated functional recovery in the animal model of multiple sclerosis^133^ and Alzheimer’s disease.^134^ HGF inhibits hyperphosphorylation of tau protein and rescues the cytoskeleton branches of damaged neurons, suggesting that MSCs-derived HGF may be the key factor for endogenous neurogenesis and cognition improvement in Alzheimer’s patients.^134^ It is worth noting that some of the growth factors secreted by MSCs support their immunoregulatory ability as well. IFNγ induces MSCs to produce flt3-ligand through the JAK/STAT signaling pathway, which binds to flt3 on CD1c^+^ dendritic cells (DCs) to promote the survival of tolerogenic CD1c^+^DCs in SLE.^135^ IGF-2, another important growth factor, is highly expressed in MSCs exposed to low oxygen and in muscle stem cells (MuSCs) and exhibits potent anti-inflammatory properties.^136^ IGF-2 administered to EAE mice preprogrammed maturing macrophages to acquire an anti-inflammatory property.^137^ It instructed maturing macrophages to undergo oxidative phosphorylation and to highly express programmed death-ligand 1 (PD-L1).^136^ However, modulation of macrophages by MSCs-derived growth factors cannot achieve the desired outcome all along. As mentioned before, PEDF is a pleiotropic protein in possession of anti-angiogenic, anti-oxidant, anti-tumor, and neuroprotective properties.^138^ PEDF interferes with macrophage infiltration and activation in the injury site, leading to a delayed remodeling process.^139^ Therefore, MSCs that highly express PEDF counteract the therapeutic actions and eventually give rise to more cardiac fibroblasts with impaired angiogenesis in the myocardial infarction region.^139^

### Inflammatory cytokines

#### Supporting cell differentiation and survival

MSCs are reported to produce a variety of factors to support the differentiation of CD34^+^ hematopoietic progenitors,^140,141^ including the cytokine IL-6. MSCs are critical for the maturation of human antibody-secreting cells (ASCs), and IL-6 is the crucial component to support the survival and secretory function of ASCs.^142^ Our previous study has shown that IL-6 derived from MSCs mediated the protective effect against the spontaneous death of splenocytes in vitro.^143^ Another report revealed that MSC(BM)s induced the proliferation of colitogenic CD4^+^ memory T cells via secreting IL-7 and played a pathogenic role in inflammatory bowel disease (IBD).^144^ IL-7 is an indispensable cytokine that contributes to B cell development, deprivation of IL-7 in MSC(BM)s reduced the tendency to differentiate into B cells of lymphoid progenitors.^3^ Moreover, LPS-triggered MSCs selectively recruit neutrophils through the secretion of IL-8 and macrophage migration inhibitory factor (MIF) to strengthen the function and survival of neutrophils.^145,146^ Considering the pro-survival property of MSCs for immune cells, the activated MSCs might boost immune responses in the injured site or exacerbate tissue necrosis under particular circumstances.^147^ Meanwhile, MSCs-derived IL-28 can trigger prostate cancer cells to undergo apoptosis, despite that IL-28 insensitive cancer cells eventually evolve in the BM.^148^ We recently showed that C3 produced by lung MSCs can promote the formation of neutrophil extracellular traps in establishing a pre-metastatic lung microenvironment. Interestingly, the C3 production by MSCs is stimulated by Th2 cytokines.^149^ Hence, the secreted factors by MSCs may serve as the essential signals to remodel the tumor microenvironment.

#### Immunoregulation and tissue remodeling

MSCs exert their immunomodulatory effects by interacting with both the innate and adaptive immune cells. MSCs reduce the expression of MHC II, CD40 and CD86 costimulatory molecules on mature DCs, as well as inhibiting the maturation of cultured DCs partially through an IL-6-dependent mechanism, thus inhibiting T-cell proliferation.^150^ Multiple cytokines are involved in the immunoregulatory functions of MSCs, including HLA-G^151^ and LIF.^152^ Splenocytes treated with MSC-CM produce large amounts of IL-10,^153^ which is an essential cytokine to induce MSCs to secrete soluble isoform of HLA-G5 to suppress innate immunity.^154^

MSCs usually perform their therapeutic function through balancing proinflammatory and anti-inflammatory responses, which are generally mediated by the suppression of excessive Th1 responses and the switch toward Th2 type. Interleukin-1 receptor antagonist (IL-1Ra) produced by MSCs is reported to alter the inflammatory and fibrotic response during chronic lung injury.^155^ IL-1Ra also can induce macrophage polarization from the M1 to M2 phenotype and accelerate wound healing.^156,157^ MSCs secreted IL-4 to polarize microglia towards the anti-inflammatory phenotype with enhanced phagocytic ability to clear extracellular α-synuclein, indicating a neuroprotective role in parkinsonian disorder.^158^ As to airway hypersensitivity mediated by uncontrolled Th2 response during asthma, MSCs diminish the content of Th2 cytokines (IL-4, IL-5, and IL-13) in bronchial lavage and Th2 type immunoglobulins in serum, through increased production of TGF-β in the activated STAT6 pathway.^159^ TGF-β in MSC-CM mediates most suppressive effects, especially for inducing regulatory T cells and inhibiting adaptive immune reactions.^160^ In LPS-stimulated microglia, TGF-β impedes their polarization to the M1 phenotype by inhibiting NF-κB signaling and restores their CX3CR1 expression, which endows them with enhanced phagocytosis of apoptotic debris.^161^ However, autocrine TGF-β in MSCs would restrict the immunosuppressive effect of MSCs via inhibiting their iNOS expression in a SMAD3-dependent manner.^162^

### ECM components

MSCs express various components of ECM, including vimentin, galectins, integrin, and collagens.^163–165^ The ECM molecules produced by MSCs are likely to support the formation and stabilization of vessels, as well as to provide ECM-associated bioactive factors.^166^ Upon the osteogenic stimuli, MSC(BM)s cultivated on collagen matrices showed decreased MMP expression along with increased tissue inhibitors of metalloproteinase (TIMPs), while the expression profile became exactly the opposite when they are subjected to adipogenic conditions.^167^ The conversion of native collagen to denatured collagen IV by MMPs is proven to switch the lineage commitment of MSCs to adipogenic differentiation.^168^ Follistatin-like protein 1 (FSTL1), a glycoprotein that has been found to mediate pro-inflammatory events, is closely correlated with chondrogenesis of MSCs, which is reflected in the production of ECM proteoglycans and collagen II.^169^

The change of ECM expression in differentiated MSCs provides a narrow window for researchers to gain insight into the communication between ECM and immune response. During the process of tissue injury and repair, chronic inflammation is always associated with aberrant ECM deposition, and the fragments of ECM may activate immune cells and support their survival during the tissue-remodeling processes.^170^ The crucial role of the ECM for adhesion and migration of inflammatory cells has been well established, which involves the HA receptor CD44 expressed by the leukocytes. The TLR3-activated MSCs strongly increased the affinity of leukocytes to MSCs through the formation of cable-like HA structures, in which the immune-suppressive activity was partially mediated by prostaglandins.^72^ In fact, MSCs co-cultured with inflammatory cells is enriched with glycocalyx, which is mainly composed of modified HA matrix, chondroitin sulfate-proteoglycan, and versican.^171^ These proteoglycans are biological macromolecules that are widely present on cell membrane surfaces and in ECM and possess a highly complex structure consisting of one or more glycosaminoglycans (GAGs) side chains with covalently conjugated core proteins. In spite of the active proteolysis in injury sites and the short life-span of active gradients, the negatively charged GAGs are usually attracted by the highly basic proteins such as chemokines, thus forming a stable structure to avoid chemokine degradation.^172^ The interaction of chemokines and GAGs is harnessed by MSCs and gives them unique advantages in quickly traversing into the circulation and mobilizing immune cells or other progenitors. As mentioned above, MSCs secreted a multifunctional extracellular component, periostin, to form a mutually reinforcing loop between matrix and B-ALL cells derived-CCL2 and increased leukemia burden.^64^ Moreover, the stable matrix structure functions as a “cloak” to make MSCs escape from host rejection and survive in xenograft transplantation. High molecular weight HA, but not that with the low molecular weight, was shown to induce IL-10-producing regulatory T cells to suppress responder cell proliferation in both human and murine system.^173,174^ In addition, MSCs constitutively secrete galectins-1 and galectins-3 that take part in T cell suppression, which also provides convincing evidence for the ECM-dependent mechanism of immunomodulation by MSCs.^164^ On the other hand, treating MSCs with fibronectin, or laminin could stimulate cell proliferation and migration,^175^ suggesting that ECM molecules further support the biological functions of MSCs. Tissue-resident HA in the kidney promoted transfused MSCs to localize in the injured renal tissue by binding with CD44 and accelerated functional recovery in an acute renal failure model.^176^ Such ECM cooperativity allows more MSCs to converge in the injury site through certain chemotaxis pathways, which may enable engineered materials to preserve active chemokines and effectively facilitate tissue repair.^177,178^

## Main pathways of MSC-mediated immunomodulation

The mechanisms that underlie the versatile immunomodulatory function of MSCs are widely described. Here, we distill some important regulatory factors that define the interface between MSCs and immune responses.

### The iNOS-NO axis

iNOS can be induced by inflammatory cytokines and is a dominant enzyme mediating the immunoregulatory effects of MSCs from rodents (such as mouse, rat, hamster, and rabbit), while MSCs from other mammalian species (such as monkey, pig, dog, cattle, and human) preferentially use IDO.^179^ The murine MSCs express high levels of iNOS upon activation by pro-inflammatory cytokines and produce NO. While IFNγ-induced NO synthesis could diminish T cell proliferation,^180^ inhibition of iNOS abolishes the mouse MSC-mediated anti-proliferative effect on T cells.^181^ Recently, we have found that the SH2 domain-containing phosphatase-1 (SHP1) negatively modulates the iNOS expression in MSCs. SHP1-deficient MSCs have higher levels of JAK1 and STAT3 phosphorylation and produce more iNOS and cyclooxygenase 2 (COX2), which endow MSCs more immunosuppressive ability in alleviating liver injury.^182^ NO may coordinate with phosphorylated STAT3 to increase PD-L1 expression in IL-17-stimulated MSCs. Thus, the IL-17 pretreated MSCs acquire more potent immunosuppressive capacity, an effect likely attributed to IL-17 modulated mRNA stability through degrading ARE/poly(U)-binding/degradation factor 1 (AUF1).^183^ However, NO is very labile and rapidly lost through oxidation. Therefore, T cells have to be attracted in close proximity to MSCs by chemokines and be restrained by adhesion molecules such as ICAM-1 and VCAM-1.^51^ During the progression of tuberculosis, the pathogen recruits MSCs to the lesion site and induces the production of NO, thereby blunting T-cell responses to help mycobacterium tuberculosis to evade host immune responses.^184^ The therapeutic efficacy of MSCs was also shown in Coxsackievirus B3 (CVB3)-induced myocarditis, indicating an important role of MSCs in antiviral immunity to blunt the cytotoxic T cell activation in a NO-dependent manner.^185^ Nevertheless, the NO-mediated immunosuppression by MSCs is likely to switch to an immune-enhancing effect under inadequate stimulus or insufficient inflammation-exposure time. Administration of iNOS inhibitor or genetic ablation of iNOS expression in MSCs could even boost immune reactions because the self-produced chemokines are still attracting immune cells.^186^ iNOS^−/−^ MSCs enhance immune responses in vitro and in vivo and suppress tumor growth as well.^186^ In addition, the antifibrotic function of MSCs-derived NO has been proposed. We have revealed that the therapeutic effect of MSCs on liver fibrosis was mediated by the expression of iNOS under inflammatory conditions. iNOS^−/−^ MSCs secreted chemokines but not NO, without any amelioration on the pathological changes in liver fibrotic mice.^74^ In the experimental model of systemic sclerosis, the iNOS^−/−^ MSCs lost the capacity of eliminating oxidative stress or exerting the anti-fibrotic effect.^187^

### The tryptophan-IDO-kynurenine-aryl hydrocarbon axis

IDO is a rate-limiting enzyme for degrading tryptophan (Trp) to N-formylkynurenine. Apart from the cell–cell contact requirement for MSC to induce T-cell tolerance, the culture medium also inhibits the proliferation of activated T lymphocytes, a paracrine effect that partly depends on the expression of IDO.^188^ The IDO-mediated conversion of Trp into kynurenine (KYN) induces apoptosis and cell cycle arrest in activated conventional T-cells and promotes the differentiation of regulatory T cells.^189^ Given that IDO-expressing macrophages suppress T-cell proliferation in vitro by reducing tryptophan concentrations,^190^ rapid exhaustion of Trp causes the generation of uncharged transfer RNA that subsequently activates the general control nonderepressible 2(GCN2) kinase, which makes T-cells unresponsive and inactive.^191^ The tryptophan catabolites such as KYN and picolinic acid could also inhibit activated T cells and NK cells in the absence of tryptophan,^192^ although the addition of tryptophan could restore allogeneic T-cell proliferation.^193^ As reported, KYN induces FOXP3^+^ Tregs in an aryl hydrocarbon receptor (AhR)-dependent manner, the binding of kynurenine to the AHR could be further potentiated by TGF-β.^194^ In human MSCs, we have found that kynurenic acid (KYNA), which is another IDO-derived metabolite with little cytotoxicity and shares the same AhR receptor as KYN, could promote TNFα-stimulated gene-6 (TSG-6) expression due to the augmented binding of AhR to the promoter of TSG-6, thereby alleviating neutrophils infiltration in injured lungs.^195^ MSCs also require IDO in promoting the differentiation of monocytes into immunosuppressive macrophages to ameliorate inflammatory responses.^196^ However, KYNA limited IL-10 production via the increase of intracellular cAMP in BM-derived macrophages and predicted poor prognosis in atherosclerosis.^197^

Human MSCs primarily express IDO upon stimulation with IFNγ together with TNFα or IL-1 to exert the immunosuppressive effects. IFNγ triggers MSCs to express IDO in a STAT1-dependent manner. STAT1 overexpression enhances MSC-mediated T-cell suppression in vitro.^198^ In addition, the pro-inflammatory stimulation leads to a metabolic shift to glycolysis. Once the glycolytic flux of MSCs is blocked by 2-Deoxy-d-glucose (2-DG) treatment, STAT1 binding to the IFNγ-activated sequence region in the IDO1 promoter is impaired, thereby abolishing IDO upregulation and reducing the inhibition on T cell response.^199^ As STAT1 phosphorylation could also be inhibited by dexamethasone, the expression of IDO or iNOS by activated MSCs would be blocked by steroids without affecting the production of chemokines.^74^ Silencing IDO in human MSCs would result in an unexpected boost of immune responses, as the MSCs could facilitate stimulated PBMC proliferation at both low and high cell densities.^186^ On the contrary, aberrant activation of IDO aggravates tumor evasion and are closely associated with poor clinical prognosis.^200^ IDO and the downstream metabolites are considered as the important mediators of MSCs to regulate immune cells, their involvement in the aging process also should be taken into account. As mentioned before, continuous inflammatory stimulation induces IDO expression but impedes MSC proliferation and differentiation. Of note, hyperactivity of IDO-mediated tryptophan degradation may be associated with a relative reduction in another metabolic pathway to generate melatonin, which serves as an antioxidant to reverse aging phenotypes of MSCs^201^ and regulate the multi-lineage differentiation of MSCs.^202^ KYN was found to be accumulated along with age in the plasma and bone tissue, making the aged mice vulnerable to bone loss and osteoporosis.^203^ These findings could be related to the observation that KYN inhibited autophagy and induced senescence in MSC(BM)s via AhR signaling.^204^

### The COX2-PGE_2_ axis

When MSCs were used to treat GvHD, it was found that MSCs displayed potent dose-dependent immunosuppressive effects on lymphocyte responses, an effect mediated by the expression of COX1/COX2 enzymes and the production of PGE_2._^205^ The immunosuppressive role of MSC(BM)s to treat EAE mice was also found to rely on PGE_2._^206^ COX2 is an inducible enzyme that mainly presents on the luminal surface of the endoplasmic reticulum and at the inner and outer membranes of the nuclear envelope.^207^ COX1, on the other hand, is encoded by a housekeeping gene and is constitutively expressed in most mammalian cells, playing a vital role in regulating renal function and protecting gastric mucosa. COX2/PGE_2_ axis has been reported as a significant mediator of MSC-mediated immune regulation, which includes programming macrophages plasticity,^208^ dampening NK cell acitivity,^209^ and suppressing Th17 differentiation.^210^ MSCs alleviate allergic inflammation by suppressing degranulation and pro-inflammatory factors production in mast cells in a COX2-dependent manner.^211^ Importantly, PGE_2_ preserves the immune privilege of allogeneic MSCs during therapeutic infusion.^212^ MSCs-derived PGE_2_ induces CD4^+^ T cell differentiation into Tregs along with TGFβ1, by means of direct cell-cell contact.^160^ In an experimental model of liver injury, PGE_2_ was found to bind to the EP prostanoid receptor 4(EP4) on CD11c^+^B220^−^ DC precursors and induce their differentiation towards a regulatory phenotype in a PI3K-dependent manner.^213^ It should be noted that COX2 is also essential for MSC-mediated tissue remodeling, especially to bone repair. COX2 could augment osteogenesis potential and suppress chondrogenic differentiation in mouse skeletal stem cells through the canonical Wnt/β-catenin signaling pathway.^214^ The COX-2/PGE2 axis plays a key role in facilitating osteogenic differentiation of MSCs in the initial pro-inflammatory phase mediated by M1 macrophages.^215^ Meanwhile, MSCs-secreted PGE2 acts on macrophages to alter the metabolic status, skewing toward M2 polarization,^208^ which is more conducive to guiding MSC differentiation and bone regeneration.

Interestingly, it has been found that hyperthermia increases the efficacy of MSC-driven immune-suppression that involves the COX2/PGE_2_ pathway, which relies on the translocation of heat shock proteins into the nucleus of MSCs.^216^ It should be noted that fever is a hallmark of inflammation and/or infection and can be triggered by PGE_2_. The COX2/PGE2 axis somehow acts to lure the inflammatory signals into cells and to activate the immunosuppressive potential of MSCs to a greater extent. For instance, when carcinoma cells-derived IL-1 increased the production of PGE_2_ in surrounding MSCs, PGE_2_ acted in concert with IL-1 to induce other cytokines, proceeding to elicit the formation of cancer stem cell niche and to promote tumorigenesis.^217^ The high amount of TNFα induces COX2 expression and PGE_2_ production in MSCs, and NO also participates in the upstream induction of COX2.^218^ Pro-inflammatory stimuli cause rapid expression of COX2 and abundant production of prostaglandins, which preferentially enter the nucleus to exert both stimulatory and inhibitory effects on the activity of NF-κB complexes, thereby promoting a series of inflammation-associated transcription.^219^ When the promoters of COX2/PTGS2 and prostaglandin E synthase (PTGES) were hypomethylated by DNA methyltransferase inhibitor, elevated production of PGE_2_ enhanced the immunosuppressive effects of MSCs on colitis mice.^220^ This COX2-based immunomodulation can also be enhanced in other ways. Phagocytosis of apoptotic cells endows human MSC(UC)s with powerful immunosuppressive capacity, the engulfment of apoptotic cells stimulates MSCs to express COX2 and produce PGE_2_ through NF-κB signaling so that it further potentiates the immunosuppressive effects of MSCs.^221^ Unlike IDO, the expression of COX2 in MSC is more variable with cell culture conditions. In vitro, the secretion of PGE_2_ by MSCs is affected by the content of fetal bovine serum (FBS) in the culture medium. The absence of FBS led to less production of PGE_2_ and compromised the immunomodulatory properties.^222^ Although hypoxia enabled MSCs to produce several growth factors and chemokines more efficiently, it accelerates proteasome-mediated degradation of COX2 and decreases PGE_2_ in MSCs, as well as loss of immune privilege.^212^ Meanwhile, arachidonic acid along with its other downstream metabolites of COX2, such as PGA_2_ and PGD_2_, displayed an inhibitory effect on IFNγ induced IDO expression in monocytes.^223^ Therefore, the interaction between PGs and the immune system cannot be generalized.

### The TNFα-TSG-6 axis

TSG-6, a 277 amino acid glycoprotein secreted by many cells in response to pro-inflammatory factors, confers MSCs with prominent anti-inflammatory properties for treating myocardial infarction,^224^ peritonitis,^225^ acute lung injury,^226^ and corneal injury.^227^ Both MSCs-secreted TSG-6 and recombinant mouse TSG-6 inhibited the STAT3 signaling pathway and alleviated pathologic changes in ethanol-induced liver injury.^228^ Our previous study showed that the IDO metabolite, KYNA, could promote TSG-6 expression by inducing the nuclear translocation of AhR and its binding to the promoter of TSG-6.^195^ The secretion of TSG-6 in MSCs was enhanced by KYNA and the protein-restricted leukocytes extravasation during inflammation.^195^ Recently, we have found that the presence of IFNγ and TNFα upregulates the expression of 11β-hydroxysteroid dehydrogenase type 1 in MSCs, subsequently augments TSG-6 expression via the canonical NF-κB pathway.^229^

The anti-inflammatory property of TSG-6 is largely due to its binding with HA fragments and the subsequent diminishment of the inflammatory network. Early in the 1990s, TSG-6 and adhesion receptor CD44 were found to share significant sequence homology, suggesting its possible binding to HA.^230^ It was later confirmed by structural analysis that the Link module of TSG-6 defines its interaction with HA.^231^ Furthermore, a part of GAGs also showed affinity to the Link_domain of TSG-6.^232,233^ It is interesting to note that the binding of Link_TSG-6 with HA is largely dependent on PH.^233^ The relative hypoxic condition and active metabolic activity of immune cells in the inflammatory sites often lead to the accumulation of metabolites such as lactic acid and contribute to the establishment of an acidic environment, which makes TSG-6 more tendentious to the damaged sites. These findings provide the molecular basis for its action mode in cell-ECM interaction and in cell migration during inflammation. In the course of cell-based therapy, TSG-6 contributes to the formation of the protective glycocalyx matrix in MSCs to circumvent xenograft rejection via the interaction with HA when exposed to inflammation.^171^ It does not only provide shelter for MSCs from host immune surveillance but also links up HA and GAGs to support the modulatory effects of MSCs. After cell transplantation, the presence of TSG-6 enables MSCs to accurately reach the damaged tissue, most likely because the microenvironment in the injured site stimulates MSCs to release more TSG-6, which may organize the surrounding HA-contained matrix complex for MSC settlement.^234^ TSG-6 also performs the immunomodulatory function by acting on immune cells. It was shown that TSG-6 inhibited activation of antigen-presenting cells and T cells in a CD44 dependent manner, consequently blocking insulitis within the pancreas and delaying the onset of type 1 diabetes.^235^ Similarly, TSG-6 reduces the TLR2/NF-κB signaling in resident macrophages in the mouse model of peritonitis through CD44.^225^ In addition, cell-to-cell contact with M1 macrophages enhanced the TSG-6 paracrine production by MSCs, which become more potent in regulating immune cells.^236^ Therefore, MSCs rely on TSG-6 to survive in transplantation and to remodel the inflammatory environment.

The inhibitory effects of TSG-6 on leukocytes transversing across vascular endothelium have been extensively investigated.^237^ Studies by the Darwin Prockop group proved that intraocular injection of recombinant human TSG-6, especially the injections within the first few hours after injury, remarkably prevented neutrophils from infiltrating in injured cornea and protected cornea from opacification and neovascularization.^227^ In fact, the mechanisms of TSG-6-mediated inhibition of neutrophil migration are more than just binding with HA.^238^ TSG-6 also modulates chemokine/GAGs interactions and then inhibits leucocytes infiltration to the damaged tissues.^239^ TSG-6 targets the GAG-binding region of CXCL8 to antagonize its interaction with heparin and prevent CXCL8-mediated neutrophil transmigration.^240^ High concentrations of TSG-6 could even obstruct the interaction of CXCL8 with its receptor CXCR2, as well as the associated neutrophil chemotaxis.^240^ TSG-6 provides the binding sites for chemokines, and the Link module of TSG-6 shows high affinities for multiple chemokines through their GAG-binding epitopes, thus disrupting the presentation of interactive chemokines on cell surfaces or their binding to collagen.^239,240^

## Advances in the application of MSC-secreted factors

**Fig. 3:**
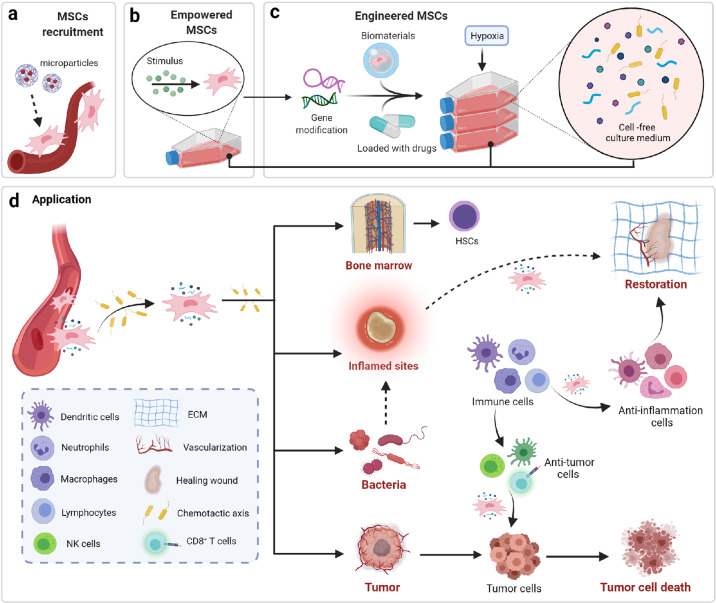
Schemes of cell/molecule-based therapy in MSCs application. The designed chemokine delivery devices have emerged as a novel approach for stem cell recruitment and tissue regeneration. Another strategy to potentiate MSCs’ secretory actions, is cultivating MSCs under low oxygen or stimulating MSCs with PRP and cytokines. Importantly, MSCs possess a unique chemotactic network to orient their transmission as the cell ark and deliver specific factors on purpose. MSCs encapsulated in biomaterials such as collagen gels or fibrous protein-based gels have increased migrating capacity to converge in damaged tissues. The factors produced by MSCs support the function and development of other cell types, such as HSC in the bone marrow. MSC also serves as a prominent vehicle to carry antibiotics to the deeply infected sites and accelerate tissue repair. The genetically modified MSCs not only achieve more homing capacity to reach target sites but also recruit more immune cells in the tumor environment to elicit an anti-tumor immunity, which bypasses the side effects caused by chemotherapeutic drugs. (Created with BioRender.com)

The cellular and molecular basis of the actions of MSC-derived factors remains to be fully elucidated, while the clinical applications of MSC-based therapy have outpaced our mechanistic understanding of their multitrophic and immunomodulatory properties (Fig. 3).

### Enhancement of secretory function

One of the most important steps for optimizing MSCs-based strategies is to stimulate MSC secretion to a greater extent. Researchers have focused on the strategy of cultivating MSCs under specific conditions of near anoxia (0.1% oxygen) to potentiate their secretory actions.^48^ Also, a few reports showed that combined administration of platelet-rich plasma (PRP) and MSCs was superior in tissue repair, especially for bone healing.^241^ Apart from the secretory organelles containing growth factors from platelets, this superiority is likely due to the transfer of mitochondria from platelets to MSCs.^242^ Such transfer could significantly augment the secretion of pro-angiogenic factors so that PRP-stimulated MSCs have improved capacity in accelerating wound healing.

### Engineered MSCs

Considering the context of pathological conditions and MSCs secretion, another option is to design cell/molecule-specific therapeutic schemes. A variety of chemokine delivery devices have emerged as a novel approach for stem cell recruitment and tissue regeneration. The designed protease-resistant chemokine CXCL12 is proven to potentiate the recruitment of CXCR4^+^/c-Kit^+^ stem cells and protect the myocardial function.^243^ The delivery of engineered alginate microparticles that contain CCL2 and VEGF is adapted for therapeutic vascularization in ischemic disease.^244^ These elements contained in biomaterials for tissue regeneration require far lower doses than the administration of unpackaged molecules. However, single agents cannot substitute all the secretory advantages of MSCs. MSCs possess a unique chemotactic network to orient their transmission as a cell ark and build positive feedback with the cells in situ. They could tactfully avoid immune rejection triggered by artificial materials, or the drug toxicity that comes from traditional pharmacological administration, so researchers are trying to take advantage of these characteristics to maximize the therapeutic potential of MSCs. It has been proven that lentiviral transduced human MSCs could persistently deliver therapeutic enzymes in vivo without affecting their trafficking ability.^245^ Silencing the gene of prolyl hydroxylase 2 (PHD2) enhances the paracrine effects of MSC(BM)s so that the modified MSCs possess a stronger ability to alleviate inflammation in necrotizing enterocolitis rats.^246^ Furthermore, engineered MSCs could serve as a prominent vehicle to carry bioactive reagents or to arise specific cellular activity on purpose. For example, translation of genetically modified MSCs that expressed α4 integrin (CD49d) achieved more bone homing in an immunocompetent mouse model, and successfully formed osteoblasts and osteocytes. The strategy may broadly benefit targeted therapies for osteoporosis.^165^ The genetically modified MSCs that highly express PEDF provide a more satisfactory outcome in preventing lung carcinoma progression.^247^ The MSCs that deliver CXCL9 and OX40 ligand, as well as the CCL19-expressing MSCs, could increase the infiltration of CCR7^+^ DCs, CD8^+^ T cells, and NK cells in tumor sites to elicit their anti-tumor effects.^248,249^ Additionally, MSC(BM)s could internalize antibiotics such as ciprofloxacin (CPX) and then release CPX to inhibit bacterial activity. Combined with the migration tendency of these cells toward the injury sites, MSCs may serve as an ideal antibiotic delivery system to convey a higher amount of antibiotics to deep infection sites.^250^ MSCs as efficient vehicles to deliver bioactive agents to the target tissues merit further exploration.

## Conclusion

MSCs are powerful bioactive agents for treating various diseases, especially for refractory immune disorders, tissue degeneration, or tissue damage, mainly through their paracrine actions. Investigations of the mediators synthesized by MSCs under various conditions should provide a better understanding of their immunoregulatory function and repair capability.
